# Supercooled preservation of cultured primary rat hepatocyte monolayers

**DOI:** 10.3389/fbioe.2024.1429412

**Published:** 2024-07-15

**Authors:** Aslihan Gokaltun, Eda Asik, Delaney Byrne, Martin L. Yarmush, O. Berk Usta

**Affiliations:** ^1^ Center for Engineering in Medicine and Surgery, Massachusetts General Hospital, Harvard Medical School, Boston, MA, United States; ^2^ Shriners Hospitals for Children, Boston, MA, United States; ^3^ Department of Chemical and Biological Engineering, Tufts University, Medford, MA, United States; ^4^ Department of Chemical Engineering, Hacettepe University, Ankara, Türkiye; ^5^ Department of Bioengineering, Hacettepe University, Ankara, Türkiye; ^6^ Department of Biomedical Engineering, Rutgers University, Newark, NJ, United States

**Keywords:** supercooled preservation, primary rat hepatocytes, 2D culture, 3-O-methyl-Α-D-Glucopyranose, polyethylene glycol, University of Wisconsin solution, hypothermosol-FRS, cold storage

## Abstract

Supercooled preservation (SCP) is a technology that involves cooling a substance below its freezing point without initiating ice crystal formation. It is a promising alternative to prolong the preservation time of cells, tissues, engineered tissue products, and organs compared to the current practices of hypothermic storage. Two-dimensional (2D) engineered tissues are extensively used in *in vitro* research for drug screening and development and investigation of disease progression. Despite their widespread application, there is a lack of research on the SCP of 2D-engineered tissues. In this study, we presented the effects of SCP at −2 and −6°C on primary rat hepatocyte (PRH) monolayers for the first time and compared cell viability and functionality with cold storage (CS, + 4°C). We preserved PRH monolayers in two different commercially available solutions: Hypothermosol-FRS (HTS-FRS) and the University of Wisconsin (UW) with and without supplements (*i.e.*, polyethylene glycol (PEG) and 3-O-Methyl-Α-D-Glucopyranose (3-OMG)). Our findings revealed that UW with and without supplements were inadequate for the short-term preservation of PRH monolayers for both SCP and CS with high viability, functionality, and monolayer integrity. The combination of supplements (PEG and 3-OMG) in the HTS-FRS solution outperformed the other groups and yielded the highest viability and functional capacity. Notably, PRH monolayers exhibited superior viability and functionality when stored at −2°C through SCP for up to 3 days compared to CS. Overall, our results demonstrated that SCP is a feasible approach to improving the short-term preservation of PRH monolayers and enables readily available 2D-engineered tissues to advance *in vitro* research. Furthermore, our findings provide insights into preservation outcomes across various biological levels, from cells to tissues and organs, contributing to the advancement of bioengineering and biotechnology.

## 1 Introduction

Preservation is a commonly used technology to maintain the viability, functionality, and structural integrity of biospecimens, such as cells, tissues, tissue-engineered products, and organs outside their natural environment over extended periods. Preservation usually involves controlling and minimizing the detrimental effects of changes in temperature, chemical agents, and other factors that can compromise the biological integrity and functionality of these specimens (Taylor et al., 2019). It plays a crucial role in tissue and organ transplantation, blood transfusion (Deller et al., 2014; Giwa et al., 2017), cell therapies (Schäffler and Büchler, 2007; Schmidt et al., 2008; Zanata et al., 2016), assisted reproduction (De Vos et al., 2014), and tissue regeneration and repair (Sodian et al., 2006; Kaita et al., 2019).

There are various approaches to preservation; the most suitable selection depends on the nature and complexity of the tissue and the required length of storage. Of these, cryopreservation preserves biological specimens in a state of suspended animation at cryogenic temperatures (−120°C to −195°C) to maintain long-term cell viability via freeze-thaw procedures and vitrification. Cryopreservation requires cell membrane-permeating (e.g., dimethyl sulfoxide) or/and nonmembrane-permeating cryoprotective agents (CPAs) such as 2-methyl-2,4-pentanediol to minimize cryoinjuries. Despite some successes at the cell level, there are still some limitations to cryopreservation. For example, cells undergo various thermal, chemical, and mechanical forces during cryopreservation, which significantly affect their biological function after preservation (Karlsson and Toner, 1996). Freeze-thaw procedures result in low cell recovery rates, and the removal of cryoprotective agents (CPAs) necessitates laborious washing and centrifugation processes (Davis et al., 1990; Karlsson and Toner, 1996; Huang et al., 2017) before further applications. Further, the presence of CPAs alters chromosome stability (Usta et al., 2013; Ullah et al., 2015), specifically when used in elevated concentration and induces spontaneous differentiation (Chetty et al., 2013), intravascular hemolysis (Meryman and Hornblower, 1972), and cell loss (Rao et al., 2015).

Hypothermic storage or cold storage (CS) is used for short-term preservation of tissues and organs at temperatures above their freezing point (*e.g.,* +4°C) (Kamijima et al., 2013; Robinson et al., 2014; Yang et al., 2017; Zhang et al., 2020; Freitas-Ribeiro et al., 2021). Under hypothermic conditions, metabolic rate and oxygen demand dramatically decrease, and this unfrozen state offers several distinct advantages compared to cryopreservation. Since CPA is not needed and phase transition does not occur, this approach provides easy harvesting, storage, and retrieval. Therefore, cryoinjuries, including osmotic shock, intracellular and extracellular ice formation, and oxidative stress, can be avoided. These advantages provide convenience at remote treatment centers and field settings, such as combat zones, where the absence of an “ultra-cold chain” is prevalent (Muraki et al., 2006; Brinkkoetter et al., 2008; Adjirackor et al., 2020; Yang et al., 2020). However, the relatively higher storage temperatures can result in irreversible cold storage-induced injuries such as adenosine triphosphate depletion, reduced enzyme activities, and extracellular matrix loss. These adverse effects ultimately lead to cell apoptosis over time. Although cold storage solutions can prolong cell survival, their effectiveness is limited, and the storage of biological samples is typically short, ranging from 6 to 48 h for cells such as human cardiac myoblasts (Abuarqoub et al., 2007), renal cells (Salahudeen et al., 2000) and hepatocytes (Duret et al., 2015; Aghdaie et al., 2020) and ranging from a few hours (*e.g.,* 4–6 h for hearts and lungs, 12 h for liver) to a few days (*e.g.,* 3 days for kidneys) (Dunn et al., 1991; Vela et al., 2018; Wang et al., 2018).

Unlike cold storage and cryopreservation, there is a possibility of exploring preservation at subzero temperatures. This alternative technology, known as supercooled preservation (SCP), lowers cellular metabolism, prevents ice formation thus, mitigates the effects of mechanical injuries and osmotic stress during cryopreservation (*i.e.,* freeze-thaw), and can potentially prolong cell survival beyond current practices of cold storage (Pegg, 2010; Taylor et al., 2019). Preventing ice formation plays a crucial role in SCP, and even a minor reduction in subzero temperatures can significantly improve organ preservation (Hertl et al., 1994) and cell survival with superior ATP levels (Matsuda et al., 1999; Usta et al., 2013). So far, SCP has been successfully employed to preserve bacterial and yeast cells (Rasmussen et al., 1975; Mathias et al., 1985), peripheral blood stem cells (Matsumoto et al., 2002), turkey spermatozoa (Zavos and Graham, 1981; Zavos and Graham, 1983), and rat hepatocytes (Matsuda et al., 1997; Matsuda et al., 1999; Almada et al., 2006; Guibert et al., 2009; Rodríguez et al., 2009; Usta et al., 2013). Recent studies have also investigated short-term organ storage via SCP of the heart (Amir et al., 2004; Monzen et al., 2005), liver (Eschwege et al., 1995; Scotte et al., 1996; Ishine et al., 1999; Soltys et al., 2001; Monzen et al., 2005; Bruinsma et al., 2015; de Vries et al., 2018; de Vries et al., 2019; de Vries et al., 2020), lung (Abe et al., 2006; Okamoto et al., 2008), and kidney (Monzen et al., 2005).

Two-dimensional (2D) engineered tissues mimic the morphological and functional characteristics of native tissues. They have been widely used in basic and preclinical biomedical research, including disease progression screening, drug testing, and drug development (Abbott and Kaplan, 2015). Despite their extensive applications, there is a lack of research on the preservation of 2D-engineered tissues that are readily usable in biomedical research. While recent studies demonstrated the preservation of monolayer rat insulinoma cells (RIN 5F) (Yamauchi et al., 2021) and a human hepatocarcinoma-derived cell line (HepG2) via SCP (Hikichi et al., 2023), the high viability of these cells is not critical since they can proliferate and cope with environmental changes *in vitro*. Consequently, there is a need for a long-term preservation solution for adherent and fragile primary cells such as hepatocytes.

Isolated liver cells, particularly isolated primary hepatocytes, have been extensively used in medicine and different fields of research, including physiological research, pharmacological testing, metabolic studies, cell transplantation, and bioartificial liver support (Tanaka et al., 2006). Still, the scarcity of fresh viable cells has hindered the utilization of hepatocytes due to the shortage of liver donors (Ostrowska et al., 2009). In successful cell isolation, it is possible to obtain −2 × 10^6^ cells/g of liver weight (Terry et al., 2006). This amount is considered ample for cell transplantation and significantly surpasses the amount required for large-scale *in vitro* assays. Thus, significant interest exists in preserving isolated hepatocytes to optimize the benefits of isolation, store large quantities of cells for transplantation, or enable accurate inter-batch comparisons for xenobiotic assays, drug detoxification, and cell therapies (Fuller et al., 2016). Several reports have been published to prolong hepatocyte as storage via cryopreservation, short-term cold storage, and supercooled preservation (Guillouzo et al., 1999; RAUEN et al., 1999; Ostrowska et al., 2000; OSTROWSKA et al., 2002; Rauen et al., 2002; Łaba et al., 2005; Terry et al., 2006; Ostrowska et al., 2009; Terry et al., 2010; Pless et al., 2012; Usta et al., 2013; Duret et al., 2015; Puts et al., 2015; Fukuoka et al., 2017; Aghdaie et al., 2020). While cryopreservation offers the advantage of indefinite storage duration, hepatocytes exhibit high susceptibility to cryoinjury, leading to significant cell loss (Guillouzo et al., 1999; Terry et al., 2006; Terry et al., 2010) and reduced cell attachment subsequently (Guillouzo et al., 1999; Terry et al., 2007). Cold storage has a protective effect by slowing down cell metabolism. Still, the cold imposes cell damage, which often worsens following the rewarming process after the preservation (RAUEN et al., 1999; Rauen et al., 2002; Rauen and de Groot, 2004). On the other hand, the preservation of hepatocytes through subzero nonfreezing temperatures prolongs the viability of these cells for much longer than cold storage. Our previous work has shown that lowering the conventional cold storage temperature of 4 C (CS) to −4.4 C (SCP) has resulted in a longer storage time of primary rat hepatocyte (PRH) cell suspensions and whole livers (Usta et al., 2013; Berendsen et al., 2014; Bruinsma et al., 2015; de Vries et al., 2018; de Vries et al., 2019; de Vries et al., 2020).

Here, we investigated how cultured PRH monolayers tolerate lower nonfreezing temperatures, SCP. We evaluated the effects of SCP at −2 and −6 C on adherent PRH monolayers and compared cell viability and functionality with conventional cold storage (CS, +4 C). To our knowledge, none of the previous reports have tested the effects of SCP on the viability and functionality of PRH monolayers. We tested two commercial baseline preservation solutions, UW and HTS-FRS, with and without supplements (*i.e.*, polyethylene glycol (PEG) and 3-O-Methyl-Α-D-Glucopyranose (3-OMG)). PRH monolayers stored in UW with and without supplements (PEG and 3-OMG) exhibit poor attachment, low viability, and functionality in plates after SCP and CS. On the other hand, the addition of supplements (PEG and 3-OMG) in the HTS-FRS solution outperforms the other groups and yields the highest viability and functional capacity. Our results demonstrated that PRH monolayers can be stored at −2 C via SCP for up to 3 days with superior viability (−62%) and functionality compared to conventional cold storage. While we focused on the SCP of PRH monolayers, our findings have broader implications for improving the preservation of cells, tissues, and organs.

## 2 Materials and methods

### 2.1 Materials

Primary rat hepatocyte culture media (C + H) was prepared with high glucose (4.5 g/L) Dulbecco’s modified eagle’s medium (DMEM; Life Technologies, CA, United States) and was supplemented with 10% fetal bovine serum (FBS, Sigma, St. Louis, MO, United States), 2% penicillin-streptomycin, 7.5 μg/mL hydrocortisone, 20 ng/mL epidermal growth factor (EGF) and 14 ng/mL glucagon. 12 well tissue culture plates were purchased from CellTreat Scientific Products (Pepperell, MA, United States). The collagen was prepared by extracting acid-soluble collagen from Lewis rat-tail tendons, as reported previously (Dunn et al., 1991). GenClone 25-508 Dulbecco’s phosphate-buffered saline (DPBS) was purchased from Genesee Scientific (Research Triangle Park, NC, United States). 3-O-Methyl-Α-D-Glucopyranose (3-OMG) was purchased from Chem-Impex Int’L Inc. (Wood Dale, IL, United States). Trypan blue, polyethylene glycol (PEG, 35 kDa), and light mineral oil (MO) were purchased from Sigma Aldrich, United States. HypoThermosol-FRS (HTS-FRS) and the Belzer University of Wisconsin (UW) were purchased from BioLife Solutions (101104, Bothell, Washington, United States) and Bridge to Life (BUW-001, Illinois, United States), respectively. Live cell imaging solution (1X), ethidium homodimer-1 (EthD-1), calcein AM and Hoechst 33342, trihydrochloride and trihydrate, and 12 and 96 well plates were purchased from Invitrogen by Thermo Fisher Scientific (Carlsbad, CA, United States). Cell counting kit-8 (CCK8) was purchased from ApexBio (Boston, MA, United States). The urea assay kit was purchased from Stanbio Laboratory (Cat. No. 0580-250). Tween 20, albumin from rat serum, o-phenylenediamine dihydrochloride tablet (10 mg substrate per tablet), hydrogen peroxide, and sulfuric acid were purchased from Sigma Aldrich (St. Louis, MO, United States). Sheep anti-rat albumin HRP conjugated (1 mL at 1 mg/mL) was purchased from Fortis Life Sciences (Waltham, MA, United States). Quick DNA miniprep kit (Cat. No. D3025) was purchased from Zymo Research (Irvine, CA, United States).

### 2.2 Primary rat hepatocyte (PRH) isolation

Primary rat hepatocytes (PRHs) were freshly isolated from 10 to 12 weeks-old adult female (180–200 g) Lewis rats (Charles River Laboratories, United States). The Cell Resource Core (CRC) performed the isolation according to protocol #2011N000111, approved by the Institutional Animal Care and Use Committee (IACUC) at Massachusetts General Hospital (MGH). Approximately 300–400 million primary rat hepatocytes were provided with 85%–95% viability, as determined via hemocytometer after trypan blue staining. The cells were seeded in 12 well plates coated with collagen immediately after isolation, as described in 2.3.

### 2.3 2D primary Rat hepatocyte monolayer culture and preservation

#### 2.3.1 Bottom collagen coating and cell seeding

12-well tissue culture plates were coated with Type I Collagen (1.25 mg/mL) before the cell seeding protocol. The collagen solution was diluted with a ratio of 1:25 in PBS at 4°C. Then, 500 µL of diluted collagen solution was added into each well and incubated at 37°C for 1 h in a humidified atmosphere with 5% CO_2_. Then, the bottom collagen gel was formed, the rest of the solution was aspirated, and wells were washed with 1 mL PBS. The cell suspension was diluted to 560,000 cells/mL (160,000 cells/cm^2^) with the C + H medium. 1 mL of this suspension was introduced to each well under sterile conditions. The plate was gently shaken via repeated vertical and horizontal movements to spread the cells evenly. Plates were incubated at 37°C with 5% CO_2_ until cells adhered to the collagen coating (40 min). The medium was then aspirated to remove dead cells, and 500 µL warm C + H medium was added into each well to refresh cell conditions. After 24 h of incubation, media were collected from each well and stored at −80°C for further functionality analyses. Then, fresh C + H media were introduced into the wells, and a cell viability assay was conducted to establish control groups and normalize the SCP data (refer to figure captions for details).

#### 2.3.2 Supercooled preservation and cold storage of PRH monolayer culture

**FIGURE 1:**
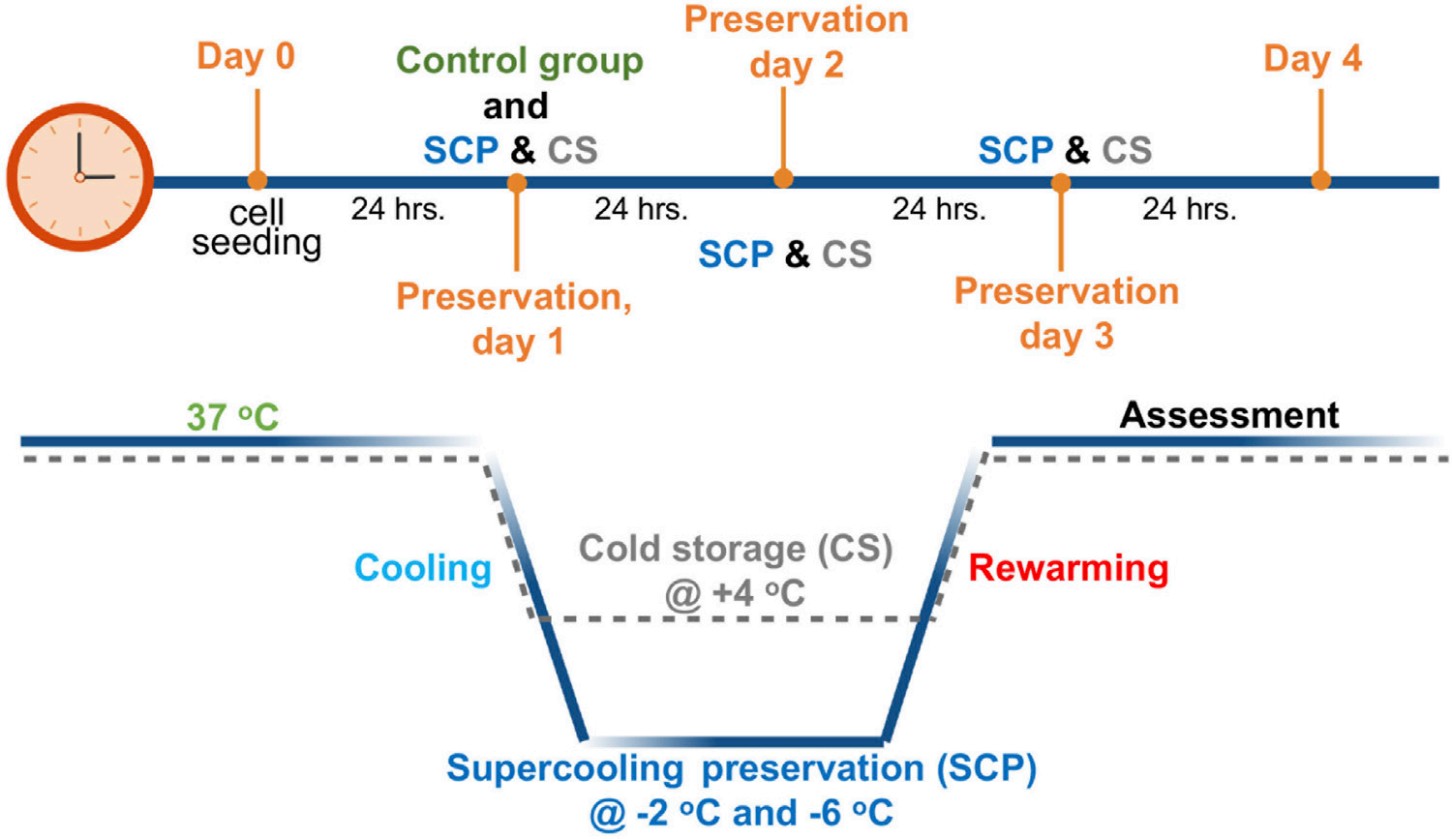
Schematic profile for supercooled preservation (SCP) and cold storage (CS). After 24 h of cell seeding, the cell culture media were removed from the wells and replaced with the 3-OMG solution for 1 h (37°C, 5% CO_2_). After 1 h of incubation, preservation solution and immiscible surface sealing agent (mineral oil) were gently added to each well, and plates were placed into temperature-controlled freezers at −2°C and −6°C for supercooled preservation. The same procedure was followed for cold storage (+4°C) of PRH monolayers without 3-OMG loading and surface sealing step. After 2 days and 3 days of preservation, cell viability and functionality were evaluated for both SCP and CS.

The supercooled preservation of PRH monolayer culture was adapted from the previously established protocol for water and suspended red blood cells (Huang et al., 2018). Briefly, 24 h after cell seeding, images were taken from each plate using an EVOS™ M5000 microscope (ThermoFisher Scientific, AMF5000, Waltham, MA) to confirm confluency. Cell culture media were removed from the wells and replaced with 500 µL 3-OMG solution (200 mM, 3-OMG dissolved in two parts distilled water and one part D-glucose-free DMEM, 310 mOsm/kg). After 1 h of incubation with 3-OMG solution (37°C, 5% CO_2_), the plates were removed from the incubator, and the solution was replaced with 1 mL of preservation solution. UW, HTS, UW supplemented with 5% w/v PEG and 200 mM 3-OMG (UW + A), and HTS supplemented only with 5% w/v PEG (shown as HTS + PEG) and 200 mM 3-OMG (shown as HTS+3-OMG) and combination of PEG and 3-OMG (shown as HTS + A) were used as preservation solutions. Finally, 1.5 mL of immiscible surface sealing agent (mineral oil) was gently added into each well -to prevent ice formation during SCP (Huang et al., 2018)- using serological pipettes, and plates were placed into portable temperature-controlled freezers (Engel MDH-13, Engel, Jupiter FL, United States) for 2 and 3 days of SCP (−2 C and −6 C) (Figure 1). These coolers were placed in cold rooms (at 4 C) to minimize temperature variations. The same procedure was followed for cold storage (CS, +4 C) of PRH monolayers, as described above, without 3-OMG loading and surface sealing steps (Figure 1).

#### 2.3.3 Cell recovery

After 2 and 3 days of CS and SCP, cell culture plates were removed from the coolers and warmed at room temperature for 15 min. The mineral oil and preservation solution were aspirated carefully, and cells were washed with 1 mL PBS at least thrice. PBS was replaced with 500 µL of warm fresh C + H media, and cells were rewarmed in an incubator (37 C, 5% CO_2_) for 2 hours before viability assays. For functionality assessments (albumin and urea secretion), media in each well was replaced with 500 µL warm, fresh C + H media. The cells are then incubated at 37 C, with 5% CO_2_ for 2 h in initial screening experiments and 24 h for the SCP and CS comparison (refer to figure captions for details). Then, media was collected from at least three wells for each experimental group and stored at −80 C for further analyses. Throughout the SCP and CS, we slowed down the metabolism of PRH monolayers for 2 and 3 days. Subsequently, after preservation, we assessed the viability and functionality and compared them with a control group that underwent incubation at 37 C for 24 h (Figure 1, control group). All groups were normalized to the control group, and their results are presented in the figures as normalized cell viability, relative albumin secretion, and urea excretion.

### 2.4 Assessment of cell viability, functionality, and attachment quantification

#### 2.4.1 Live dead staining and CCK-8 assay

After cell recovery, cell viability was determined using a standard live/dead assay kit and CCK-8 assay. The fluorescent dye calcein AM and ethidium homodimer were utilized to evaluate the cellular esterase activity and membrane integrity of the cell membrane through fluorescence imaging. The staining solution was prepared by mixing 10 mL live cell imaging solution (1X), 5 µL calcein AM reagent, 2 µL Hoechst 33342, and 20 µL ethidium homodimer-1. Cell media were aspirated, and the wells were washed with 1 mL of Live cell imaging solution (1X). 1 mL of staining solution was added into wells, and the plate was incubated at 37 C, 5% CO_2_ for 20 min. Then, the staining solution was replaced with 1 mL of the cell imaging solution, and viability was evaluated with the EVOS™ M5000 microscope. Viable cells were identified by the exclusion/minority of ethidium homodimer (red) and the retention of calcein (green), whereas cells stained with ethidium homodimer (red) were counted as non-viable. CCK-8 assay was also utilized to quantify cell viability. For control groups, after 24 h of initial cell seeding, 50 µL of CCK-8 was added into each well containing 500 µL of C + H medium and incubated at 37 C, 5% CO_2_ for an hour. For SCP samples, after cell recovery, 50 µL of CCK-8 was added into each well containing 500 µL of fresh C + H medium, and the plates were incubated at 37 C, 5% CO_2_ for an hour. Later, 80 µL samples were collected from each well in a 96-well plate, and the absorbance values were measured using a microplate reader (Molecular Devices, SpectraMax^®^ ID3) at 450 nm.

#### 2.4.2 Albumin analysis

Albumin secretion of PRH monolayers was determined using an in-house enzyme-linked immunosorbent assay (ELISA) protocol. Substrate buffer (5.1 g citric acid and 7.29 g sodium phosphate dibasic in 1 L distilled water, pH 5), 8N sulfuric acid (22.2 mL concentrated sulfuric acid in 77.8 mL distilled water), PBS-Tween 20 solution (0.05 v/v% Tween 20 in PBS) and OPD solution (mixing one pill of OPD, 25 mL substrate buffer, and 10 µL of 30% H_2_O_2_) were prepared before analysis. First, 96 well plates were coated with 100 µL of 50 μg/mL rat albumin. Then, the plate was sealed with a plate sealer and incubated at 4 C overnight. Next, the plate was washed four times with PBS-Tween 20 solution. 50 μL of samples or standards were added to wells in triplicate. Following, 50 µL of antibody solution (1:10000 dilution in PBS-Tween) was added to each well. The plate was covered with an adhesive plate sealer and incubated for 1.5 h at 37 C. After incubation, the plate was washed with PBS-Tween 20 solution four times, and then 100 µL of OPD solution was added to the wells at regular intervals. Finally, the reaction was stopped by adding 50 µL 8N sulfuric acid (H_2_SO_4_) to each well. The absorbances of each well were then measured using a microplate reader (Molecular Devices, SpectraMax^®^ ID3) at 490–650 nm. Standard curves were created for each plate, and albumin concentrations were calculated correspondingly.

#### 2.4.3 Urea analysis

Urea excretion of PRH monolayers was analyzed using a Stanbio Urea BUN assay kit following the protocol by the manufacturer. Briefly, the urea assay reagent was prepared by mixing one part BUN color reagent with two parts BUN acid reagent. 10 μL of samples or standards were pipetted into a 96-well plate in triplicate. 150 μL urea reagent was added to each well with a multichannel pipet. The plate was sealed tightly and incubated at 60 C for 90 min. Then, the plate was removed from the incubator and cooled down at room temperature for 10 min. The absorbance values were read at 540 nm using a microplate reader (Molecular Devices, SpectraMax^®^ ID3).

#### 2.4.4 DNA extraction

The DNA quantity of PRH monolayers in each well was determined using a Quick-DNA Miniprep Kit (Zymo Research, D3024) following the manufacturer’s protocol. Isolated DNA quantity was used to normalize the viability and functionality data for the supercooled preservation (SCP) and cold storage (CS) comparison. This approach was used to mitigate potential inconsistencies across wells (refer to figure captions for details). Briefly, the cell culture medium was initially aspirated, and cells were lysed by adding a Genomic Lysis Buffer in each well. Then, samples were transferred to a Zymo-Spin™ IICR Column. After consecutive washing and centrifuging steps with DNA-Pre-Wash Buffer and g-DNA-Wash Buffer, respectively, DNA was eluted from the samples via DNA Elution Buffer. The DNA concentrations of the samples were measured using a nanodrop (Thermo Scientific, NanoDrop One).

#### 2.4.5 ImageJ analysis

The relative cell attachment area of PRH monolayers after 2 and 3 days of supercooled preservation (SCP) at −6 C in UW, UW + A, HTS, and HTS + A was quantified using phase contrast images in ImageJ. All images converted into an 8-bit format, cell-attached areas were marked and measured using freehand selections. The total cell attached area was then calculated. Each group’s attachment area (UW, UW + A, HTS, HTS + A) was normalized to the cell attachment area of fresh control groups.

### 2.5 Statistical analysis

Origin Pro 2021 Graphing & Analysis Software v.9.0.8.200 (Origin Lab, Northampton, Massachusetts) was used to analyze data. All quantitative data were presented as the mean ± standard error of the mean (SEM) from three different wells (n = 3) and three different isolations (N = 3). The statistical significance of the results was assessed using one-way ANOVA with Tukey multiple comparisons. Statistical significance was defined as *p* < 0.05 for all experiments.

## 3 Results

We examined two commercially available solutions for the supercooled preservation of PRH monolayers with and without supplements: 1) HTS-FRS (labeled as HTS herein) and 2) UW, which have been used for the preservation of primary hepatocytes in the previous reports (Adams et al., 1995; Arikura et al., 2002; Janssen et al., 2003; Meng, 2003; Sosef et al., 2005; Mathew et al., 2006; Ostrowska et al., 2009; Usta et al., 2013; Gramignoli et al., 2014; Jorns et al., 2014; Duret et al., 2015). As supplements, we used PEG (35 kDa, 5% (w/v)) as an extracellular cell membrane stabilizer (Oltean et al., 2012; Puts et al., 2015) and 3-OMG (200 mM) as a nonmetabolizable intracellular cytoprotectant (Sugimachi et al., 2006) either alone or together. All experiments were conducted with PRH monolayers in 12 well plates.

**FIGURE 2:**
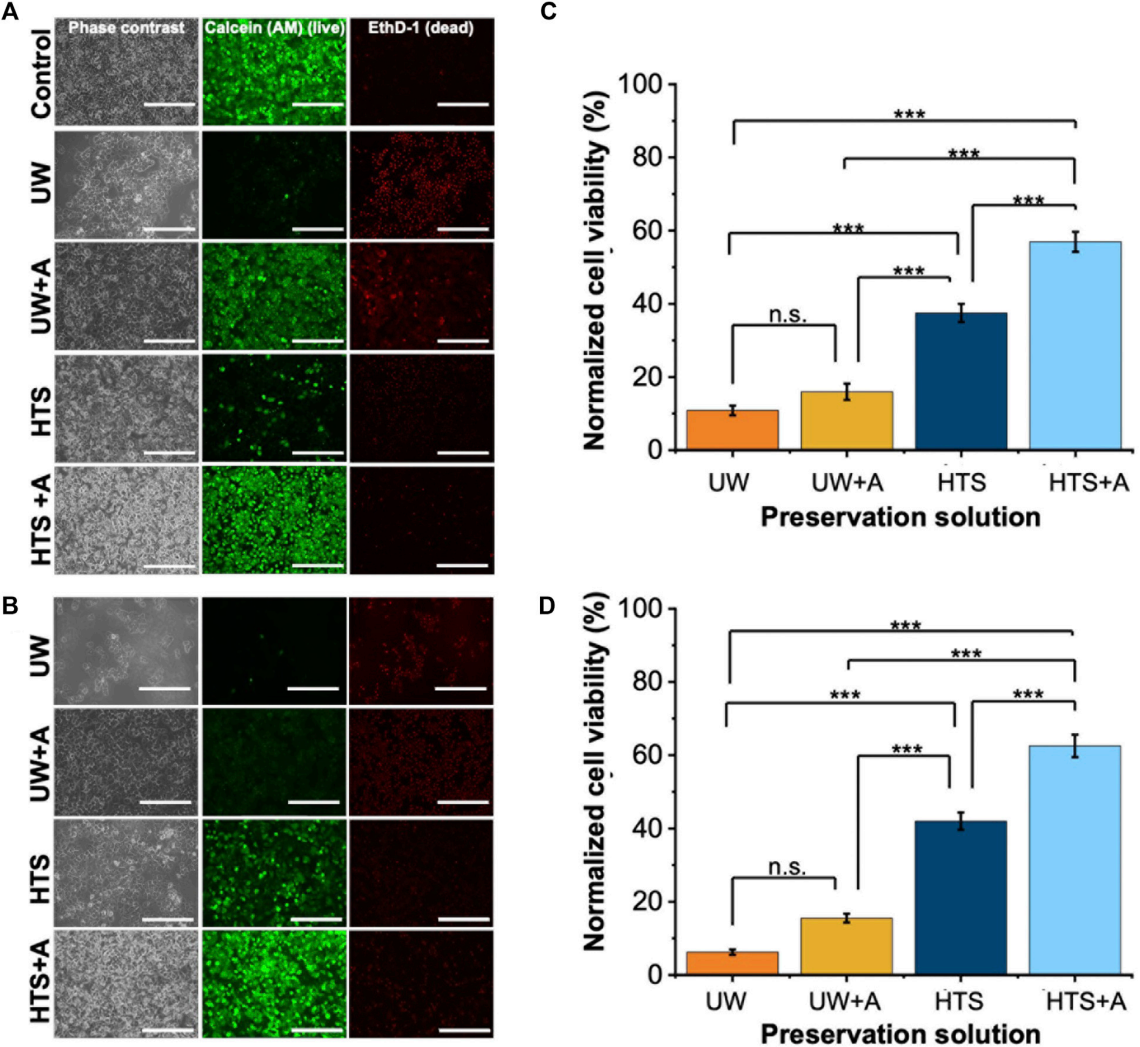
Cell staining and normalized cell viability comparison after 2 and 3 days of SCP of PRH monolayers (−6°C) with different preservation solutions. UW + A: UW supplemented with 5% w/v polyethylene glycol (PEG) and 200 mM 3-O-methyl glucose (3-OMG). HTS + A: HTS supplemented with 5% w/v polyethylene glycol and 200 mM 3-O-methyl glucose (3-OMG). PRH monolayers were treated with the 200 mM 3-OMG solution for an hour (37°C, 5% CO_2_) before SCP. Cells were stained by calcein AM (live) and ethidium homodimer (dead) after **(A)** 2 and **(B)** 3 days of supercooled preservation. Normalized cell viability after **(C)** 2 and **(D)** 3 days of SCP at −6°C. The CCK-8 cell viability assay was conducted 24 h after initial cell seeding, serving as controls. All groups were normalized to the control group. The HTS + A group has significantly higher viability than UW, UW + A, and HTS on both days. According to staining images **(A, B)** and cell viability results **(C, D)**, the HTS + A group had significantly higher viability than UW, UW + A, and HTS on both days. Cell viability was assessed via CCK-8 assay. The viability was determined after 2 h of rewarming in cell culture media (C + H) at 37°C. Three different isolations were conducted, and three different wells from each isolation were stained (N = 3, n = 3). CCK-8 cell viability data are expressed as mean ± SD (n = 3, N = 3). We use n.s.: non-significant, and ***≤ 0.001 by Tukey-method for significance comparisons between groups. Scale bar: 200 µm.

We initially investigated the effect of preservation solution on cell viability by conducting a) live/dead staining (Figures 2A,B, Supplementary Figures S1, S2) and CCK-8 cell viability assay (Figures 2C,D) following SCP of PRH monolayers for 2 and 3 days at −6°C. Staining images indicated that PRH monolayers preserved in UW presented low cell viability on both days (Figures 2A,B). They presented 11% and 6% viabilities via CCK-8 assay after 2 and 3 days of SCP, respectively (Figures 2C,D). Although the cells were confluent and fully attached to the well plates before SCP, PRH monolayers preserved in UW could not maintain their monolayer integrity and detached by −40% and −80%, respectively, from the bottom of the wells after 2 and 3 days of SCP (Supplementary Figures S3A–C). The addition of supplements (either PEG (shown as UW + PEG), 3-OMG (shown as UW+3-OMG), or a combination of PEG and 3-OMG in UW (shown as UW + A) enhanced cell attachment and maintained monolayer integrity better than UW alone. Nevertheless, the cell viability of PRH monolayers preserved in UW + A was still low on days 2 and 3 (−15%) (Figures 2A–D).

In contrast, PRH monolayers preserved in HTS retained their monolayer integrity better than UW after 2 and 3 days of SCP (Figures 2A,B, Supplementary Figures S2, S3). The addition of supplements in HTS, either PEG (5%, 35 kDa) and 3-OMG (200 mM) or a combination of both (shown as HTS + A) enhanced preservation outcomes at −6°C after 2 days SCP (Supplementary Figure S2). PEG and 3-OMG improved post-storage confluency and viability compared to HTS alone, but their effects on viability were higher when used together (Supplementary Figures S3A, B). Moreover, the combination of supplements provided enhanced cell attachment (over 90%) after 2 and 3 days of SCP (Supplementary Figure S3C). The viability of PRH monolayers preserved in HTS + A was around 63% after 3 days of SCP (Figure 2D). Thus, our findings demonstrated that the combination of supplements was necessary and had a significant impact when used with HTS to improve cell viability at −6°C for 2 and 3 days of SCP. We also extended the SCP to 4 days, preserving the PRH monolayers in base solutions with supplements (5% PEG and 200 mM 3-OMG) and evaluated the cell viability using live/dead staining (Supplementary Figure S4). We observed that PRH monolayers lost their viability when stored in UW + A and HTS + A after 4 days of SCP (Supplementary Figure S4).

**FIGURE 3:**
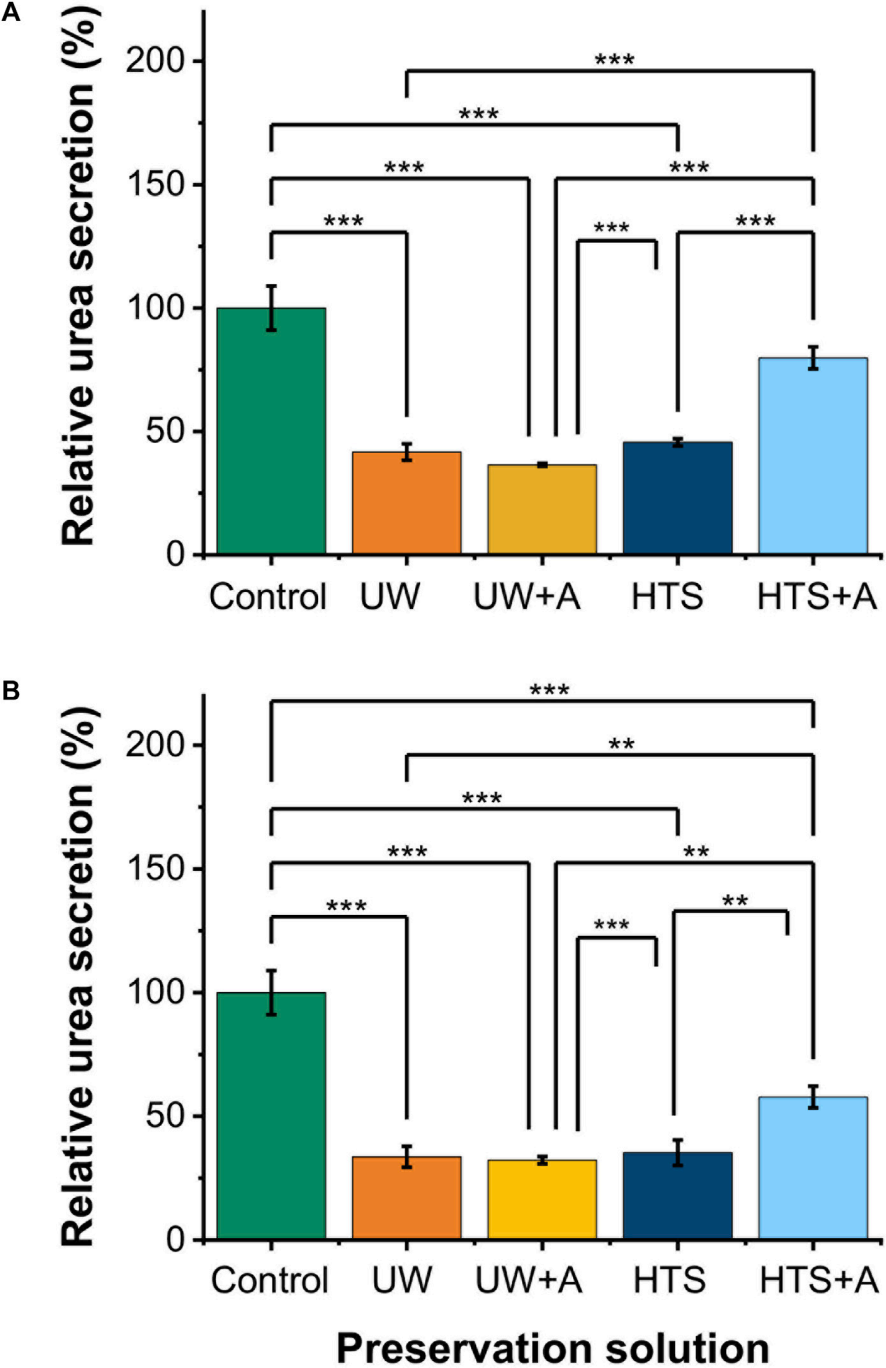
Relative cell functionality comparison of 2 and 3 days SCP of PRH monolayers (−6°C) at different preservation solutions. UW + A: UW supplemented with 5% w/v polyethylene glycol (PEG) and 200 mM 3-O-methyl glucose (3-OMG). HTS + A: HTS supplemented with 5% w/v polyethylene glycol and 200 mM 3-O-methyl glucose (3-OMG). PRH monolayers were treated with the 200 mM 3-OMG solution for an hour (37°C, 5% CO_2_) before SCP. Urea excretion of cells after **(A)** 2 and **(B)** 3 days of supercooled preservation at −6°C. The urea excretion in the HTS + A group is statistically higher than in UW, UW + A, and HTS groups on days 2 and 3. Average urea excretion in the control group: 28.1 μg/mL/2 h/0.56 M cells. Albumin and urea samples were collected and analyzed 24 h after SCP. All groups were normalized to the control group. Data are expressed as mean ± SD (n = 3, N = 3). We use **≤ 0.01 and ***≤ 0.001 by Tukey-method for significance comparisons between groups.

Next, we assessed the functionality of PRH monolayers after 2 and 3 days of SCP at −6°C (Figures 3A,B). While PRH monolayers preserved in UW, UW + A, and HTS, showed statistically similar urea excretions, HTS + A stood out with significantly higher urea excretions than other preservation solutions (Figures 3A,B) on both days. Together with cell viability, SCP of PRH monolayers at −6°C with HTS + A solution exhibited better viability and functionality than other preservation solutions.

To compare supercooled preservation and cold storage of PRH monolayers outcomes, we then moved to evaluate the differences between UW, HTS, and HTS + A solutions for cold storage (4°C) of PRH monolayers after 3 days of preservation and following 2 h of rewarming in cell culture media (C + H) (Supplementary Figure S5). Almost all the PRH monolayers preserved in UW were detached from the wells after 2 and 3 days of CS (Supplementary Figures S6A,B). UW supplemented with either 5% w/v PEG (shown as UW + PEG) and 200 mM 3-OMG (shown as UW+3-OMG) and a combination of 5% w/v PEG and 200 mM 3-OMG (shown as UW + A) did not enhance monolayer integrity even after 2 days of CS (Supplementary Figure S6C). PRH monolayers preserved in HTS and HTS + A demonstrated better viability than UW (Supplementary Figure S5A). We observed that the addition of supplements (PEG and 3-OMG) in HTS did not significantly affect the cell viability as indicated in staining images and cell viability assay results (Supplementary Figure S5); PRH monolayers preserved in HTS and HTS + A showed almost similar viabilities (40% and 46%, respectively) after 3 days of preservation at +4 C (Supplementary Figure S5B). Accordingly, we continued quantifying functionality using better performing preservation solutions, HTS and HTS + A (Supplementary Figure S7). Albumin secretion was statistically similar in PRH monolayers preserved in HTS and HTS + A. However, higher urea excretion was observed for PRH monolayers preserved in HTS. Interestingly, while the addition of supplements in HTS did not significantly affect the cell viability after 3 days of cold storage, PRH monolayers preserved in HTS + A were able to maintain much better functionality than HTS (Supplementary Figure S7).

**FIGURE 4:**
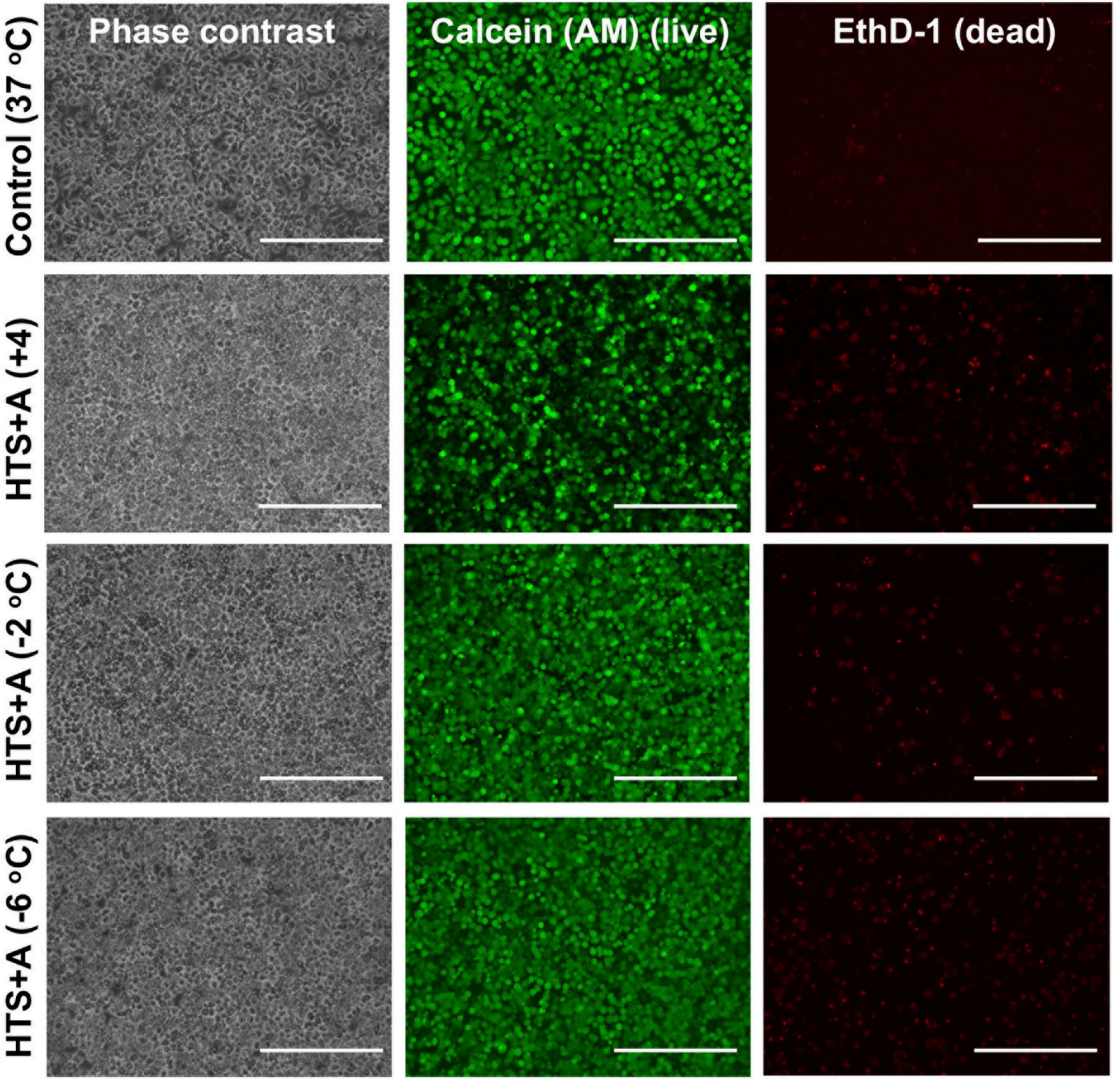
Cell staining comparison of SCP and CS of PRH monolayers in HTS + A at different temperatures. The staining of live cells and dead cells was achieved by calcein AM and ethidium homodimer, respectively. The staining was performed following 2 h of rewarming in cell culture media (C + H) at 37°C. According to staining images, SCP groups at −2°C and −6°C represented higher viability compared to the CS group at +4°C. Three different isolations were conducted, and three different wells from each isolation were stained (N = 3, n = 3). Scale bar: 200 µm.

**FIGURE 5:**
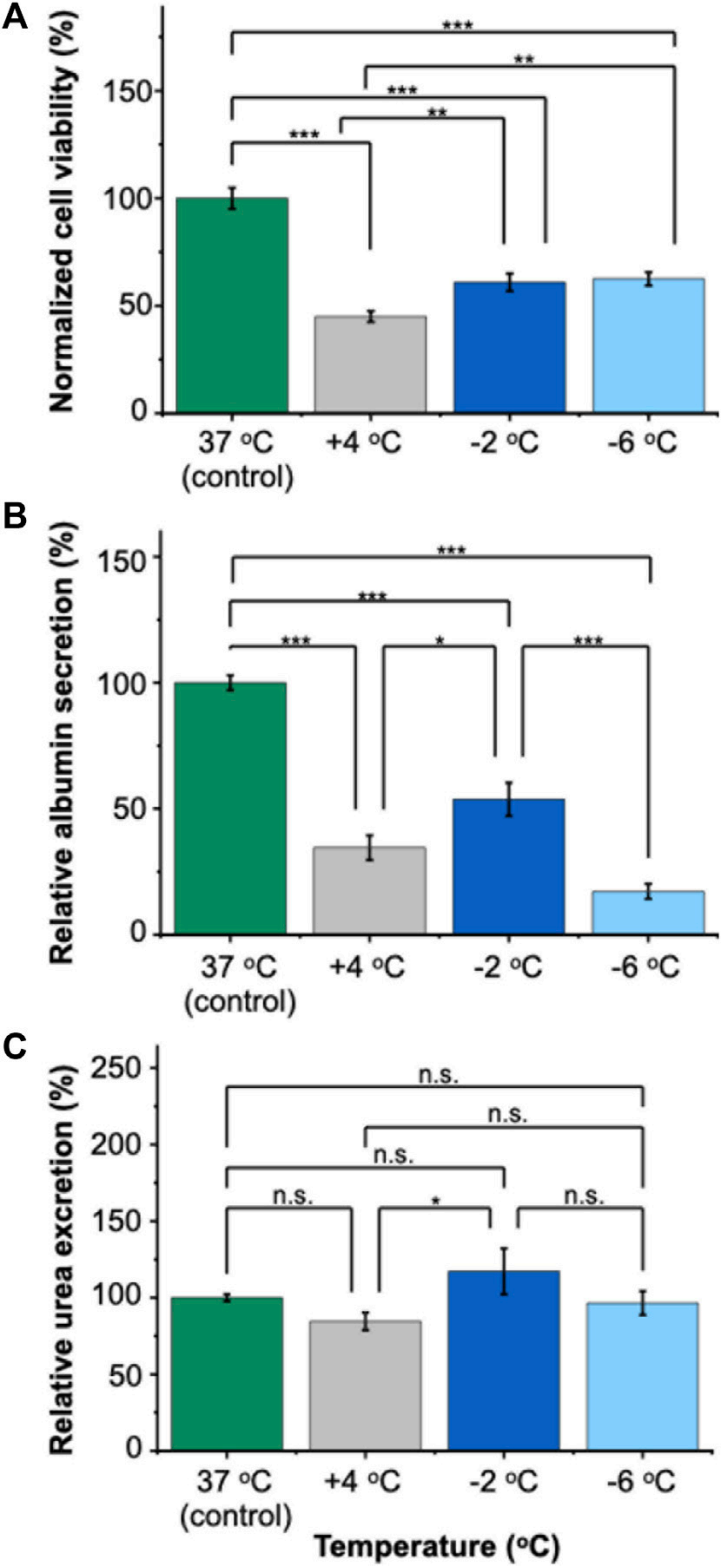
Relative viability and functionality comparison of 3 days of SCP and CS of PRH monolayers in HTS + A at different temperatures. **(A)** Cell viability after 3 days of SCP and CS. The −2°C and −6°C groups had significantly higher viability than cold storage (+4°C). The viability was determined following 2 h of rewarming in cell culture media (C + H) at 37°C, **(B)** albumin secretion of cells at different temperatures. The albumin secretion in the −2°C group is statistically higher than +4°C and −6°C. Average albumin secretion in the control group: 26.8 μg/mL/24 h/0.56 M cells, **(C)** urea excretion of cells after 3 days of CS. The −2 °C group had significantly higher urea excretion than the +4°C group. The average urea excretion in the control group was 171.2 μg/mL/24 h/0.56 M cells. PRH monolayers were treated with the 200 mM 3-OMG solution for an hour (37°C, 5% CO_2_) before SCP. Albumin and urea samples were collected and analyzed 24 h after SCP. All groups were first normalized to the DNA content within each well, followed by normalization to the control group. Data are expressed as mean ± SD (n = 3, N = 3). We use n.s.: non-significant, *≤ 0.05, **≤ 0.01, and ***≤ 0.001 by Tukey-method for significance comparisons between groups.

Our results demonstrated that PRH monolayers preserved in HTS + A outperformed other groups in terms of viability and functionality both for SCP and CS. Thus, using the best-performing preservation solution, HTS + A, we compared the viability and functionality of PRH monolayers after 3 days of SCP and CS (Figures 4, 5). We used three different groups -*i.e.*, PRH monolayers preserved at **a)** −2°C, **b)** −6°C, and **c)** +4°C and normalized our findings with the control group at 37°C. Based on the staining images and CCK-8 cell viability assay, SPC of PRH monolayers at both temperatures (−2°C and −6°C) resulted in significantly higher cell viability compared to CS (+4°C) (Figures 4, 5A). PRH monolayers preserved at −2°C outperformed other groups regarding functionality (Figures 5B,C). PRH monolayers preserved in HTS + A at −2°C showed the highest albumin secretion and urea excretion compared to other groups, while PRH monolayers preserved at +4°C and −6°C were statistically similar in albumin secretion and urea excretion.

## 4 Discussion

Mammalian cells are typically cryopreserved in suspension, but cells are used as adherent monolayers for basic or preclinical research (e.g., disease progression toxicity screening). This creates a mismatch between the storage format, requiring significant time and expertise for cell preparation. Cryopreservation of cells directly in plates could streamline the process, allowing cells to be used after preservation and facilitating high-throughput screening. However, current methods have limitations to cryopreserve cells in the monolayer format, which results in low cell recovery rates (<30%) (Pless-Petig et al., 2018). Intracellular ice formation is a particular challenge for the cryopreservation of cells in monolayers, as cell-cell contacts promote the propagation of ice, contributing to low recovery rates (Acker et al., 1999; Acker and McGann, 2000; Acker et al., 2001; Higgins and Karlsson, 2013). Various methods have been explored to reduce intracellular ice formation, yet it still remains challenging (Bahari et al., 2018; Daily et al., 2020; Murray et al., 2022).

In this study, we aimed to preserve PRH monolayers via SCP to create “ready-to-use” monolayers for research. We tested the differences between two commercially available solutions -UW and HTS-FRS (labeled as HTS herein) for SCP. Previous reports tested both solutions for cryopreservation, CS, and SCP of hepatocyte suspensions (Adams et al., 1995; Arikura et al., 2002; Janssen et al., 2003; Meng, 2003; Sosef et al., 2005; Mathew et al., 2006; Ostrowska et al., 2009; Usta et al., 2013; Gramignoli et al., 2014; Jorns et al., 2014; Duret et al., 2015). Additionally, we supplemented the base solutions with PEG (35 kDa, 5% (w/v)) as an extracellular cell membrane stabilizer (Oltean et al., 2012; Puts et al., 2015) and 3-OMG (200 mM) as a nonmetabolizable intracellular cytoprotectant (Sugimachi et al., 2006) either alone or together. We investigated their effect on PRH monolayer viability and functionality after SCP. We then compared our results with cold storage.

Hepatocytes constitute approximately 80% of the liver’s total mass and are crucial for detoxification and activation of xenobiotics and endogenous molecules (Gomez-Lechon et al., 2010; Olsavsky Goyak et al., 2010). As such, the liver removes ammonia, a toxic byproduct of glutamine metabolism, by converting it into urea via the urea cycle (Arias et al., 2020). Therefore, urea excretion serves as a vital indicator of liver functionality. Accordingly, in our initial screening experiments, we selected the best-performing solution based on viability and urea excretion (Figures 2, 3). We included albumin secretion in subsequent experiments.

HTS-FRS and UW solutions primarily aim to maintain intracellular balance. Their composition varies in ionic concentrations, impermeants, and the use of different buffers (Ostrowska et al., 2009). The UW solution contains a high concentration of potassium ions (K^+^), whereas the HTS-FRS solution contains a high concentration of sodium ions (Na^+^) (Serrar et al., 1999; Ostrowska et al., 2009; Pless-Petig et al., 2012). Additionally, these two solutions have slightly different osmotic pressures: −320 mOsm/L (Serralta et al., 2005) for UW and −350 mOsm/L for HTS-FRS. Nevertheless, previous reports showed that osmolarity had minimal effects on the cold storage of PRHs (Pless-Petig et al., 2012). One notable distinction among these solutions is the antioxidant composition, which is crucial in reducing the levels of reactive oxygen species generated during preservation. While the UW solution contains glutathione and allopurinol, the HTS-FRS solution contains vitamin E analogs “Trolox” and glutathione to diminish damage and consequent initiation of apoptosis effectively. It was shown that Trolox had a high iron-chelating capacity (Li et al., 2011), which prevented damages beyond 48 h (Ostrowska et al., 2009) and was identified as crucial in facilitating the attachment ability of the PRH monolayers after cold storage (Li et al., 2011; Pless-Petig et al., 2012). Unlike HTS, PRH monolayers preserved in UW significantly detached (40% and 80%) after 2 and 3 days of SCP respectively (Figures 2A,B, Supplementary Figure S3). Thus, cell detachment might likely be due to the absence of the potent antioxidant -Trolox- in UW. Another reason could be the inadequate anti-oxidant additive (glutathione) content in UW solution for the preservation of cells beyond 24 h, as it has a half-life of 1 day (Evans et al., 1996).

To further improve cell viability and functionality, the preservation solution can be supplemented with membrane stabilizers, antioxidants, and energy substrates. PEG is known for its positive effects on cell and organ preservation (Cheng et al., 2012) and its molecular weight (MW) plays a significant role. For example, low MW PEG (5 kDa) was observed to accumulate in hepatocytes (Mack et al., 1991). MWs ranging from 0.4 to 20 kDa were identified to activate JNK signaling, which could exhibit either protective or harmful effects (Dutheil et al., 2009). Moreover, PEG MWs ranging from 0.4 to 20 kDa interacted with the membrane glycerophospholipids (Dutheil et al., 2006). On the other hand, PEG with a MW of 35 kDa has demonstrated several advantages in the preservation of cells and organs, including antioxidant activity ability to prevent edema and membrane stabilization and freeze protection in subzero preservation conditions (Mack et al., 1991; Bhatnagar et al., 2010; Oltean et al., 2012; Berendsen et al., 2014; Puts et al., 2015). Despite its benefits, PEG’s inability to penetrate the cell membrane differs from the natural mechanisms observed in freeze-tolerant wood frogs, where freezing prompts a rapid breakdown of liver glycogen to produce a large amount of glucose. This glucose is then circulated throughout the frog’s body, contributing to the preservation of structural integrity in the frog’s cells and organs during freezing (Storey, 1987; King et al., 1993; Storey and Mommsen, 1994). Based on this, some reports investigated the effectiveness of a non-metabolizable glucose derivative, 3-O-methyl glucose (3-OMG), for the preservation of mammalian cells. Previous research demonstrated that 3-OMG was transported into the cell via members of the GLUT family and can accumulate within the cytoplasm at an appreciable level (Longo et al., 1988). Once internalized, 3-OMG was metabolically inert and nontoxic for mammalian cells (Longo et al., 1988; Sugimachi et al., 2006). Studies on primary rat hepatocytes loaded with 3-OMG (200 mM, a total osmolarity of 310 mOsm/kg) demonstrated high post-thaw viability and preserved long-term hepatospecific functions (Sugimachi et al., 2006). Moreover, studies also confirmed 3-OMG’s efficacy in SCP of the liver (Sugimachi et al., 2006; Berendsen et al., 2014). Thus, to improve SCP, we also incorporated a nontoxic, intracellular protectant, 3-OMG, before and during preservation. Our results indicated that the addition of supplements in UW, either alone or in combination, improved post-storage confluency but did not significantly enhance viability compared to UW alone. (Supplementary Figure S1). Moreover, neither PEG nor 3-OMG alone sufficiently increased the viability of PRH monolayers in HTS for SCP (Supplementary Figure S2). However, when combined in HTS, PEG and 3-OMG improved monolayer viability compared to other SCP groups (Supplementary Figures S1, S2) and CS (Supplementary Figure S5). This enhancement is likely due to the necessity of intracellular and extracellular protectants during SCP of PRH monolayers. Conversely, for CS of PRH monolayers, the combination of supplements did not significantly improve viability compared to HTS (Supplementary Figure S5). We speculate that this stems from differences in injury mechanisms at SCP and CS temperatures, where we expect higher membrane related injuries for SCP (Usta et al., 2013; Puts et al., 2015).

In terms of functional capacity, during SCP, although PRH monolayers displayed comparable viabilities at both −2°C and −6°C, a notable decrease in albumin secretion was observed at −6°C (Figure 5B). This decrease could potentially be explained by the increasing membrane injuries of PRH monolayers at decreasing preservation temperatures (*i.e.,* −6°C). The irreversible injuries to cell membranes likely happen at these low temperatures (*i.e.,* −6°C), which would then manifest as loss of membrane integrity and, subsequently, the direct lysis of the cells or a slow apoptosis response eventually. This was despite our theoretical expectation that lower temperatures would further slow metabolism (Liu et al., 2014; Huang et al., 2018) and improve preservation outcomes. Additionally, we observed the urea excretion exceeded 100% in some cases during SCP and CA (Figure 5C, Supplementary Figure S7B). This heightened excretion might be due to changes in cellular metabolism (e.g., glutamine metabolism (Arias et al., 2020)) or altered metabolic pathways, necessitating further investigation. Among all SCP and CS groups, HTS + A at −2°C is the most optimal choice regarding viability and functionality (*i.e.,* albumin secretion, Figure 5B). Our results indicated that achieving viable SCP of PRH monolayers with minimal loss of function requires both lowering the preservation temperature below CS (+4°C) and supplementing the preservation solution with PEG and 3-OMG.

## 5 Conclusion and future outlook

In this study, we investigated the preservation of PRH monolayers using SCP and compared our results with CS regarding viability and functionality. We used two commercially available solutions (UW and HTS-FRS) with and without supplements (PEG and 3-OMG). Our findings demonstrated the significance of maintaining a non-frozen, supercooled state to extend the storage time of PRH monolayers. We demonstrated that the choice of preservation solution is critical, and the addition of PEG and 3-OMG to the HTS solution is essential for maintaining higher viability and functionality of the PRH monolayers during SCP. Specifically, our results indicated that HTS performed better than UW for SCP and CS as a base preservation solution for PRH monolayers. Furthermore, HTS supplemented with PEG and 3-OMG (HTS + A) improved cell viability, attachment, and functionality of PRH monolayers for SC. In contrast, these supplements did not significantly affect the viability and functional capacity of PRH monolayers in CS. Importantly, although PRH monolayers showed almost similar viability at −2°C and −6°C after 3 days of SCP, −2°C was the optimal storage temperature for maintaining the highest functional capacity (*i.e.,* albumin secretion).

The enhanced cell viability and functionality of PRH monolayers through supercooled preservation (SCP) is a significant advance toward high-quality preservation of engineered tissue models. Still, there is room for further improvement and novel strategies—the use of novel antioxidants, membrane stabilizers, and energy substrates before, during, and after preservation—can be instrumental for such improvement. Additionally, it is important to identify which family of genes (*e.g.,* extracellular matrix and adhesion molecule genes or mitochondrial metabolism genes) are altered before and after SCP at different times. Such deeper molecular understanding can provide more profound insights into subzero non-freezing biology and have far-reaching implications for improving preservation outcomes for cells, tissues, and organs. Such advancements in preservation technologies will transform cell-based therapies, streamline *in vitro* drug screening studies, and enhance the efficiency of organ transplantation logistics.

## Data Availability

The original contributions presented in the study are included in the article/Supplementary Material, further inquiries can be directed to the corresponding author.
