# Supplementary material for: Supercooled preservation of cultured primary rat hepatocyte monolayers
Source: Front Bioeng Biotechnol. 2024 Jul 15;12:1429412. doi: 10.3389/fbioe.2024.1429412 (PMC11284110; doi:10.3389/fbioe.2024.1429412)
Supplement: Supplementary file 1 [file DataSheet1.doc]

**Supercooled Preservation of Cultured Primary Rat Hepatocyte Monolayers Supporting Information**

**Figure S1. Cell staining comparison after 2 days of supercooled preservation (SCP) of PRH monolayers at -6 ^o^C.** The monolayers were preserved in UW and UW supplemented with either 5% w/v PEG (shown as UW+PEG) and 200 mM 3-OMG (shown as UW+3-OMG) and a combination of 5% w/v PEG and 200 mM 3-OMG (shown as UW+A). According to staining images, the addition of supplements in UW, either alone or together, improved post-storage confluency but did not significantly improve viability compared to UW alone. PRH monolayers were treated with the 200 mM 3-OMG solution for an hour (37 ^o^C, 5% CO_2_) before SCP. Cells were stained by calcein AM (live) and ethidium homodimer (dead) after 2 days of SCP. The viability is determined following 2 hours of rewarming in cell culture media (C+H) at 37 ^o^C. Three isolations were conducted, and three wells from each isolation were stained (N=3, n=3). Scale bar: 200 µm.


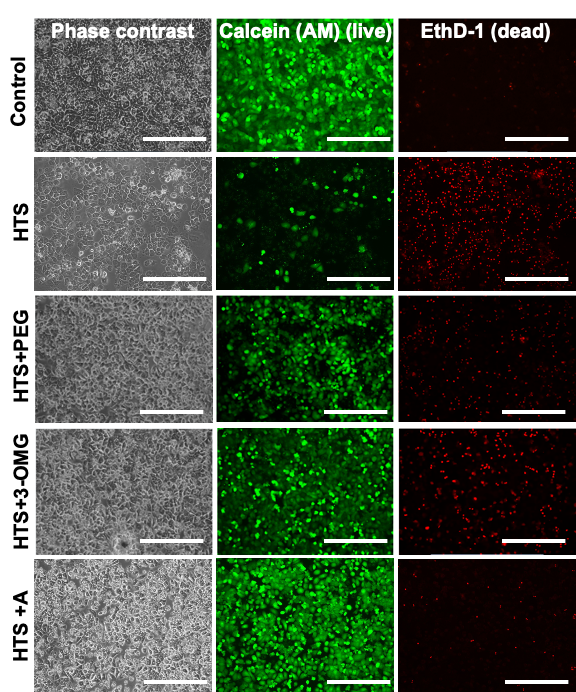


**Figure S2. Cell staining comparison after 2 days of supercooled preservation (SCP) of PRH monolayers at -6 ^o^C.** The monolayers were preserved with HTS and HTS supplemented with either 5% w/v PEG (shown as HTS+PEG) and 200 mM 3-OMG (shown as HTS+3-OMG) and a combination of 5% w/v PEG and 200 mM 3-OMG (shown as HTS+A). According to staining images, both PEG and 3-OMG improved post-storage confluency and viability compared to HTS alone, but their effects on viability were higher when they were used together. PRH monolayers were treated with the 200 mM 3-OMG solution for an hour (37 ^o^C, 5% CO_2_) before SCP. Cells were stained by calcein AM (live) and ethidium homodimer (dead) after 2 days of SCP. The viability is determined following 2 hours of rewarming in cell culture media (C+H) at 37 ^o^C. Three isolations were

conducted, and three wells from each isolation were stained (N=3, n=3). Scale bar: 200 µm.

**Figure S3.** **Phase contrast images and the relative cell attachment area of PRH monolayers after 2 and 3 days of supercooled preservation (SCP) at -6 ^o^C in UW, UW+A, HTS, and HTS+A.** Panel **a)** represents the phase contrast images of cells before and 2 days after SCP, **b)** represents the phase contrast images of cells before and 3 days after SCP, and **c)** shows the relative cell attachment area of PRH monolayers after SCP. Each group (UW, UW+A, HTS, HTS+A) was normalized to the cell attachment area of fresh control groups. PRH monolayers were confluent and attached to the well plates before SCP. After 2 and 3 days of SCP in UW, PRH monolayers could not maintain their monolayer integrity and detached by 40% and 80%, respectively, from the bottom of the wells. The addition of supplements (5% w/v PEG and 200 mM 3-OMG) to both UW and HTS enhanced cell attachment and maintained monolayer integrity better during SCP. PRH monolayers were treated with the 200 mM 3-OMG solution for an hour (37 ^o^C, 5% CO_2_) before SCP. The relative cell attachment area was calculated using the phase contrast images in Figures S3 and S4 with Image-J.


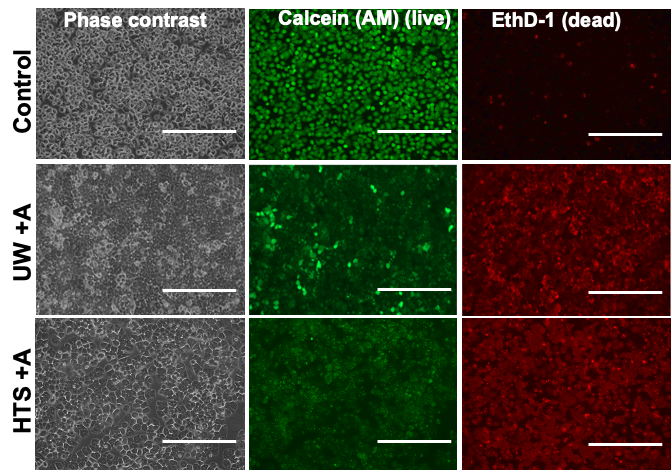


**Figure S4: Cell staining images after 4 days of supercooled preservation (SCP) of PRH monolayers in UW+A and HTS+A.** According to staining images, both groups did not keep their viability 4 days after SCP. PRH monolayers were treated with the 200 mM 3-OMG solution for an hour (37 ^o^C, 5% CO_2_) before SCP. Cells were stained by calcein AM (live) and ethidium homodimer (dead). The viability was determined following 2 hours of rewarming in cell culture media (C+H) at 37 ^o^C. Three isolations were conducted, and three wells from each isolation were stained (N=3, n=3). Scale bar: 200 µm.

**
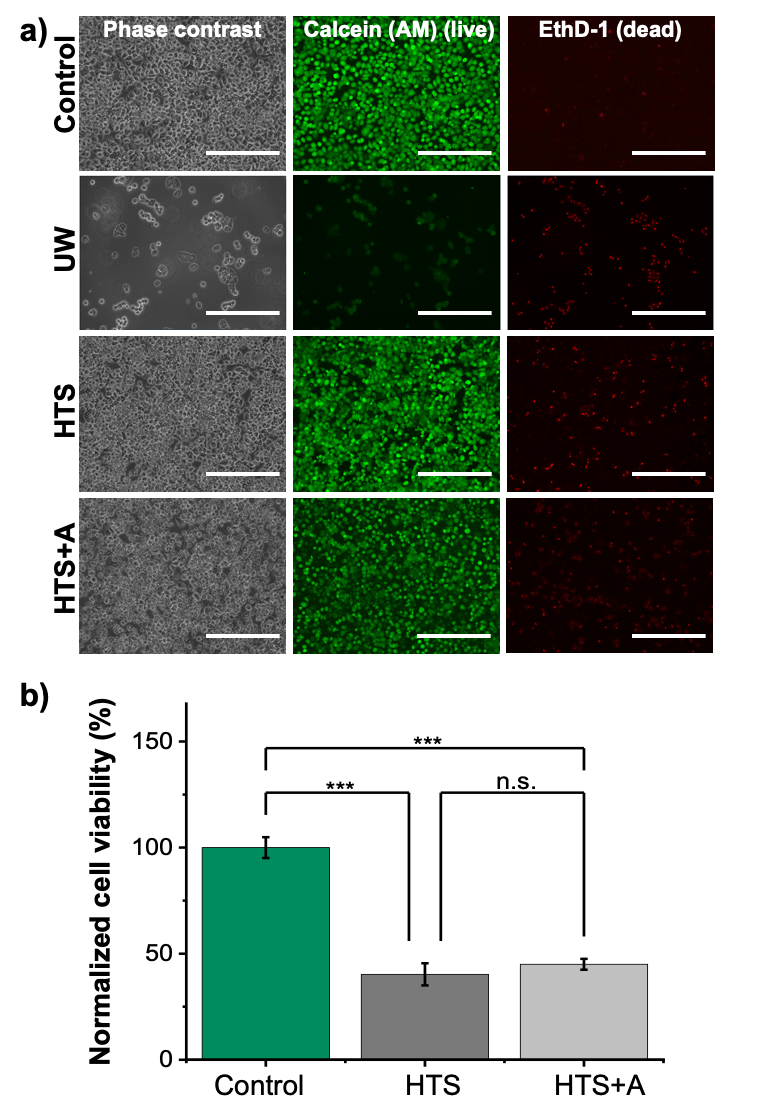
**

**Figure S5.** **Cell staining and relative cell viability comparison after 3 days of cold storage (CS, +4 ^o^C) of PRH monolayers with different preservation solutions.** UW, HTS, and HTS supplemented with polyethylene glycol (PEG, 5% w/v) and 3-O-methyl glucose (3-OMG, 200 mM) (HTS+A) were tested. The staining of live cells and dead cells was achieved by calcein AM and ethidium homodimer, respectively. The staining and viability are determined following 2 hours of rewarming in cell culture media (C+H) at 37 ^o^C. After CS, almost all of the PRH monolayers preserved in UW detached from the wells. The HTS and HTS+A group has higher viability compared to UW. According to the results of staining images and cell viability, the HTS and HTS+A groups have statistically similar viability. Three isolations were conducted, and three wells from each isolation were stained (N=3, n=3). Cell viability was assessed via CCK-8 assay. All groups were first normalized to the DNA content within each well, followed by normalization to the control group. CCK-8 cell viability data are expressed as mean ± SD (n = 3, N = 3). We use n.s: non-significant, and ***≤ 0.001 by Tukey-method for significance comparisons between groups. Scale bar: 200 µm.

**Figure S6.** **Phase contrast images of PRH monolayers a) before and after 2 days of CS in UW, b) before and after 3 days of CS in UW, c) before and after 2 days of CS in UW supplemented with either 5% w/v PEG (shown as UW+PEG) and 200 mM 3-OMG (shown as UW+3-OMG) and a combination of 5% w/v PEG and 200 mM 3-OMG (shown as UW+A).** The PRH monolayers were confluent and fully attached to the well plates before CS; however, they could not maintain their monolayer integrity even with supplements, and they were detached from the bottom of the wells. The phase contrast images were taken following 2 hours of rewarming in cell culture media (C+H) at 37 ^o^C. Scale bar: 200 µm.

**Figure S7. Comparison of cell functionality after 3 days of cold storage (CS, +4 ^o^C) of PRH monolayers with different preservation solutions**. **a)** albumin secretion of PRH monolayers after 3 days of cold storage. The albumin secretion is statistically similar with HTS and HTS+A, Average albumin secretion in the control group: 26.8 µg/mL/24 hr./0.56 M cells, and **c)** urea excretions of PRH monolayers after 3 days of cold storage. The HTS group has significantly higher urea excretion than HTS+A. The average urea excretion in the control group is 171.2 µg/mL/24 hr./0.56 M cells. Albumin and urea samples were collected and analyzed 24 hours after CS. All groups were first normalized to the DNA content within each well, followed by normalization in reference to the control group. Data are expressed as mean ± SD (n = 3, N = 3). Scale bar: 200 µm. We use n.s.: non-significant, and **≤ 0.01 by Tukey-method for significance comparisons between groups.
